# A8 LUMINAL SHORT CHAIN FATTY ACIDS INCREASE ACTIVITY IN COLONIC MYENTERIC PLEXUS NEURONS IN MICE

**DOI:** 10.1093/jcag/gwad061.008

**Published:** 2024-02-14

**Authors:** P Wongkrasant, W MacNaughton, K Sharkey

**Affiliations:** University of Calgary, Calgary, AB, Canada; University of Calgary, Calgary, AB, Canada; University of Calgary, Calgary, AB, Canada

## Abstract

**Background:**

The enteric nervous system (ENS) plays a critical role in the control of gastrointestinal functions. Short chain fatty acids (SCFAs) are the products of non-digestible dietary fiber fermentation by gut microbial metabolism and are a primary energy source for enterocytes. However, the impact of SCFAs on the ENS is not well understood.

**Aims:**

We aimed to test whether SCFAs affect enteric neuronal function using a novel intact preparation of the colon.

**Methods:**

Intact segments of the colon from mice expressing a genetically encoded fluorescent calcium (Ca^2+^) reporter specifically in all enteric neurons (Wnt-1-GCaMP6 mice) or in intrinsic primary afferent neurons (IPANs, Calb1-GCaMP6 mice) were bathed and luminally perfused with Krebs solution. Contractility was inhibited by nicardipine (3 µM) and scopolamine (1 µM). The duration and intensity of neuronal intracellular Ca^2+^ fluorescence responses to SCFA were visualized by live-cell confocal microscopy and were analyzed using Imaris™ software. The SCFAs were luminally perfused at a constant rate of 10 mL/h for 3 min. NaCl was modified in Krebs solution to correct for the osmolarity of SCFAs.

**Results:**

In the myenteric plexus of Wnt-1-GCaMP6 mice, SCFAs (acetate, 60 mM; propionate, 20 mM or butyrate, 20 mM) caused a rapid and sustained increase in Ca^2+^ fluorescence (26.8±5.9, 25.8±1.7, and 27.4±5.9 min, respectively). The peak Ca^2+^ responses were 92.5%± 28.3%, 122.6%±21.5% and 111.7%± 23.0%, respectively. The combination of these three SCFAs increased Ca^2+^ fluorescence for 20.5±6.3 min and potentiated levels of Ca^2+^ fluorescence to 125.1%±21.6% above baseline. The percentages of neurons responding to SCFAs were 85.7%, 94.4%, 97.7% and 93.3 % for acetate, propionate, butyrate and a combination of SCFAs, respectively.

In Calb1-GCaMP6 mice, IPANs showed a similar pattern of Ca^2+^ responses and a proportion of neurons that responded to the SCFAs. Following acetate, propionate, butyrate and combination of SCFAs administration, fluorescence was increased for 13.5±1.2, 20.6±2.7, 24.9±2.2 and 46.1±4.6 min, by a magnitude of 404.3%±153.7%, 129.0%±0.4%, 73.9%±18.8% and 717%±267.0% above baseline and the percentages of neuronal response were 100.0%, 100.0%, 100.0% and 87.5 %, respectively.

**Conclusions:**

We show that SCFAs activate neurons of the myenteric plexus including IPANs. These studies suggest that some of the actions of SCFAs are mediated by the ENS, but further studies are needed to identify the underlying mechanisms of action of SCFAs in the myenteric plexus.

**Funding Agencies:**

CAG, CIHRTRIANGLE Canada

